# Correction: Silva et al. Chemical Profile and Bioactivities of Extracts from Edible Plants Readily Available in Portugal. *Foods* 2021, *10*, 673

**DOI:** 10.3390/foods11060845

**Published:** 2022-03-16

**Authors:** Beatriz Nunes Silva, Vasco Cadavez, Pedro Ferreira-Santos, Maria José Alves, Isabel C. F. R. Ferreira, Lillian Barros, José António Teixeira, Ursula Gonzales-Barron

**Affiliations:** 1 CEB—Centre of Biological Engineering, University of Minho, 4710-057 Braga, Portugal; beatrizsilva@ceb.uminho.pt (B.N.S.); pedrosantos@ceb.uminho.pt (P.F.-S.); jateixeira@deb.uminho.pt (J.A.T.); 2 Centro de Investigação de Montanha (CIMO), Instituto Politécnico de Bragança, Campus de Santa Apolónia, 5300-253 Bragança, Portugal; vcadavez@ipb.pt (V.C.); maria.alves@ipb.pt (M.J.A.); iferreira@ipb.pt (I.C.F.R.F.); lillian@ipb.pt (L.B.)

The authors found a mistake in the original paper [1]. Throughout the article, French lavender was wrongfully named rosemary. Rosemary was not used in the original study. Below are provided the full details of the changes in the Figures, Tables, and text. The authors sincerely apologise for any inconvenience caused and state that the scientific conclusions are unaffected. The original publication has been updated.

## 1. Errors in Figures

In the original publication, there was a mistake in the Graphical Abstract as published. “Rosemary” should be replaced by “French lavender” in the legend of the plot. The corrected Graphical Abstract appears below.

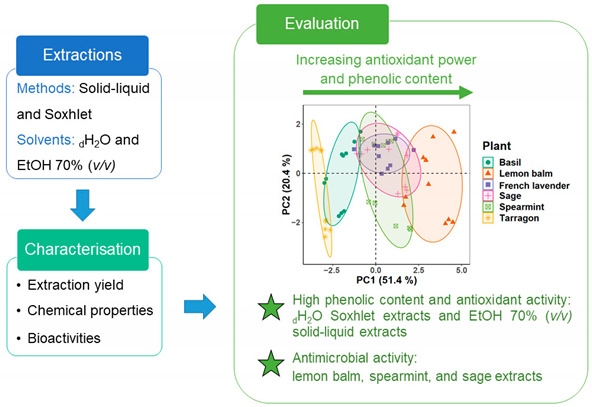



In the original publication, there was a mistake in Figure 2 (page 7) as published. “Rosemary” should be replaced by “French lavender” in the legend of plot C. The corrected Figure 2 appears below.

In the original publication, there was a mistake in Figure 3 (page 10) as published. “Rosemary” should be replaced by “French lavender”. The corrected Figure 3 appears below.

## 2. Errors in Tables

In the original publication, there was a mistake in Table 1 (page 9), Table 2 (page 13) and Table S2 (Supplementary Materials), as published. “Rosemary” should be replaced by “French lavender”. The corrected Table 1, Table 2 and Table S2 appear in the following pages.

## 3. Text Corrections

In the Abstract, line 2, the word “rosemary” should be revised to “French lavender”.

On page 2, Section 1, third paragraph, line 7, the word “rosemary” should be revised to “French lavender” and the references should be updated from [3,7–9] to [3,7–11] to include another two references. The updates to the references are further explained in the section References below.

On page 2, Section 1, third paragraph, line 8, citations [10,11] should be changed to [12,13].

On page 2, Section 1, fourth paragraph, line 4, citation [12] should be changed to [14].

On page 2, Section 1, fifth paragraph, line 4, citations [13,14] should be changed to [15,16].

On page 2, Section 1, sixth paragraph, citation [15] was replaced with a new one, [10] (see below, in References) and the following correction has been made:

“In folk medicine, French lavender (*Lavandula stoechas* L.) is a well-known aromatic plant that has been used for its anti-inflammatory, antispasmodic and carminative properties, as well as for its positive effects against various problems, including eczema, urinary tract infections and heart-burn, for example [10].

On page 2, Section 1, seventh paragraph, line 3, citation [16] should be changed to [17].

On page 2, Section 1, seventh paragraph, line 5, citation [17] should be changed to [18].

On page 2, Section 1, eighth paragraph, line 3, citation [18] should be changed to [11].

On page 2, Section 1, eighth paragraph, line 5, citation [18] should be changed to [11].

On page 2, Section 1, tenth paragraph, line 2, the word “rosemary” should be revised to “French lavender”.

On page 2, Section 2.1, first paragraph, line 1, the word “rosemary” should be revised to “French lavender”.

On page 5, Section 3, first paragraph, line 1, the word “rosemary” should be revised to “French lavender”.

On page 8, Section 3.2, fifth paragraph, line 1, the word “rosemary” should be revised to “French lavender”.

On page 10, Section 3.3, fifth paragraph, line 6, the word “rosemary” should be revised to “French lavender”.

On page 14, Section 3.4, fifth paragraph, line 2, the word “rosemary” should be revised to “French lavender”.

On page 14, Section 3.4, seventh paragraph, line 3, the word “rosemary” should be revised to “French lavender”.

On page 14, Section 3.4, eighth paragraph, one citation was added (new reference [52]), former citation [52] should be changed to [53] and the following correction has been made:

“Other researchers have also studied the phenolic profile of the plant materials used in our work. Nunes et al. performed the characterization of phenolic compounds from *L. stoechas* L. methanolic extracts [52]. Their research identified rosmarinic, ferulic, chlorogenic and vanillic acids, among other compounds. From our French lavender extracts, rosmarinic, ferulic and chlorogenic acids were also detected (the latter in only one of the extracts), while vanillic acid was never detected. Zgórka and Głowniak studied the phenolic profile of sage, basil and lemon balm extracts [53]. Their work indicated the presence of vanillic acid in sage and basil (approximately 25 and 6 μg/g dry plant, respectively). In our study, vanillic acid was detected in the aqueous solid–liquid sage extract at 250 μg/g dry plant (12.4 mg/L extract) and in two basil samples obtained by solid–liquid extraction at 260 and 340 μg/g dry plant (12.9 and 17.2 mg/L extract). Their research also revealed the existence of ferulic acid in sage (around 50 μg/g dry plant); and rosmarinic acid (the most predominant compound) in basil, lemon balm and sage (approximately 11650, 9690 and 5120 g/g dry plant, respectively). Our study also identified ferulic acid in all sage extracts, and rosmarinic acid in all basil, lemon balm and sage extracts. Zgórka and Głowniak did not identify chlorogenic acid in any of the tested plant extracts, which was also the case in our study, depending on the extraction method and solvent used [53].”

On page 14, Section 3.4, ninth paragraph, citation [53] should be changed to [54] and the following correction has been made:

“Kivilompolo et al. also performed the characterization of phenolic acids from sage, basil and spearmint extracts [54]. Their research identified rosmarinic acid in basil, spearmint and sage (3080, 5620 and 9960 μg/g dry plant)—like we did in our study—as well as chlorogenic acid in basil; vanillic acid in sage and spearmint; and syringic, p-coumaric and ferulic acids in all herb extracts. With some exceptions, most of these outcomes agree with those presented in Table 2. In contrast, Kivilompolo et al. [54] reported the presence of vanillic acid in basil (140 μg/g dry plant) and chlorogenic acid in sage and spearmint (230 and 310 μg/g dry plant). In our study, vanillic acid was only detected in two basil extracts, as previously referred; chlorogenic acid was never identified in sage, and only detected in one spearmint extract (180 μg/g dry plant; 8.77 mg/L extract).”

On page 15, Section 3.4, tenth paragraph, line 3, citation [54] should be changed to [55].

On page 15, Section 3.4, tenth paragraph, line 4, citation [55] should be changed to [56].

On page 15, Section 3.4, eleventh paragraph, line 1, citations [54,55] should be changed to [55,56].

On page 15, Section 3.4, twelfth paragraph, line 3, citation [55] should be changed to [56].

On page 15, Section 3.4, thirteenth paragraph, line 5, citation [53] should be changed to [54].

On page 16, Section 3.5, third paragraph, line 2, citation [56] should be changed to [57].

On page 16, Section 3.5, fifth paragraph, line 1, citation [57] should be changed to [58].

On page 16, Section 3.5, fifth paragraph, line 6, citation [58] should be changed to [59].

On page 16, Section 3.5, sixth paragraph, line 1, citation [59] should be changed to [60].

On page 17, Section 3.5, sixth paragraph, line 4, citation [60] should be changed to [61].

On page 17, Section 3.5, seventh paragraph, line 4, citation [60] should be changed to [61].

On page 17, Section 3.5, seventh paragraph, line 5, citation [61] should be changed to [62].

## 4. References

One reference has been replaced and renumbered ([15] is now [10]); one reference has been renumbered ([18] is now [11]); and one reference has been added (new reference [52]). The numbering throughout the article has been updated accordingly.

Please see below:

Replaced and renumbered:

The previous reference [15] has been renumbered and replaced by:

10. Ez zoubi, Y.; Bousta, D.; Farah, A. A Phytopharmacological review of a Mediterranean plant: *Lavandula stoechas* L. *Clin. Phytoscience* **2020**, *6*, 9.

Renumbered:

The previous reference [18] has been renumbered:

11. Alu’datt, M.H.; Rababah, T.; Alhamad, M.N.; Ereifej, K.; Al-Mahasneh, M.; Brewer, S.; Rawshdeh, M. Optimization extraction conditions for phenolic compounds, antioxidant and inhibitory activities of angiotensin-I converting enzyme (ACE), α-glucosidase and α-amylase from *Mentha spicata* L. *J. Food Biochem.* **2015**, *40*, 335–344.

Added:

52. Nunes, R.; Pasko, P.; Tyszka-Czochara, M.; Szewczyk, A.; Szlosarczyk, M.; Carvalho, I.S. Antibacterial, antioxidant and antiproliferative properties and zinc content of five south Portugal herbs. *Pharm. Biol.* **2017**, *55*, 114–123.

## Figures and Tables

**Figure 2:**
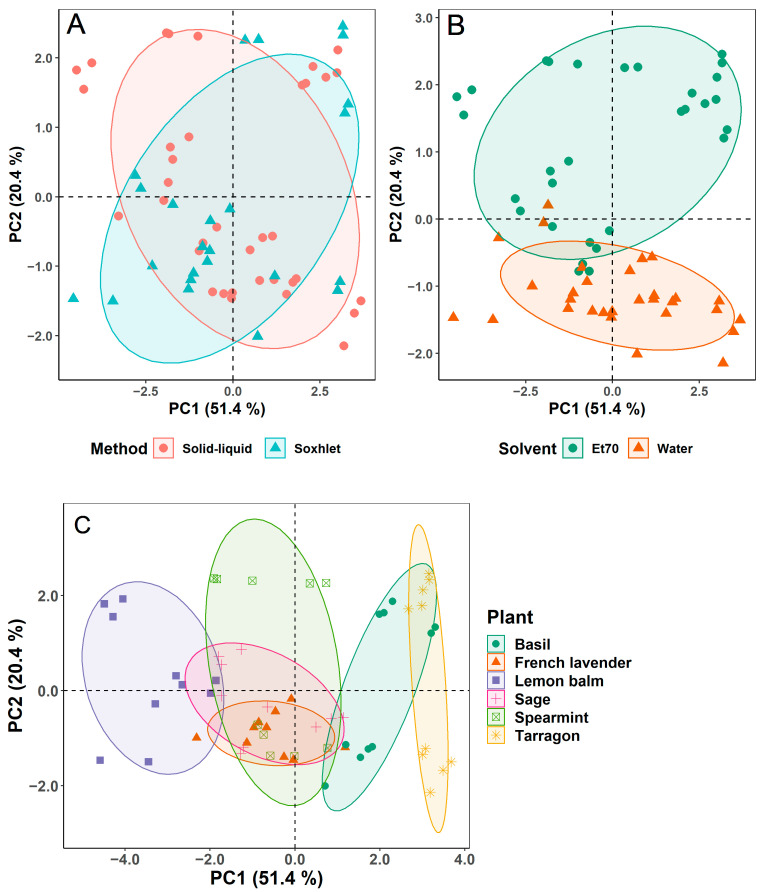
Score plots of the first two components of the principal component analysis (PCA) grouped by extraction method (**A**), solvent (**B**) and plant type (**C**).

**Figure 3:**
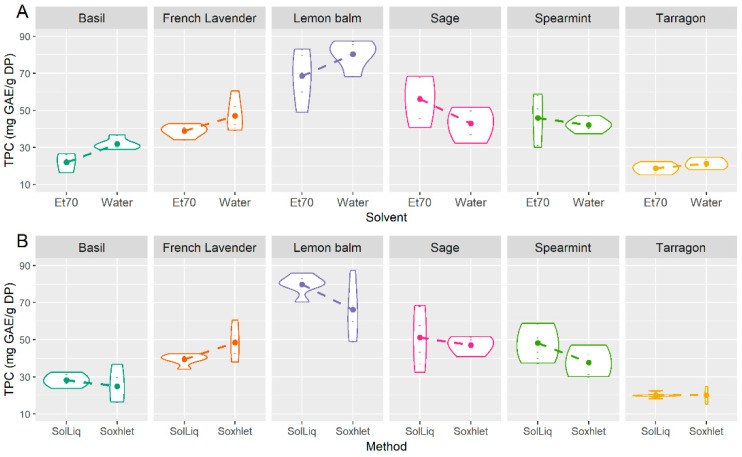
Interaction plots “plant × solvent” (**A**) and “plant × method” (**B**) on the total phenolic content of plant extracts.

**Table 1:** Groupwise summary statistics (mean ± standard error) by plant, method, and solvent, for each chemical characterization and antioxidant assay, and significance of the main effects and interactions of the models.

	Yield (%)	Ch-a (μg/g DP)	Ch-b (μg/g DP)	TProtein (μg BSAE/g DP)	TFC (mg CE/g DP)	TPC (mg GAE/g DP)	Carbohydr. (μg GE/g DP)	DPPH (μmol TE/g DP)	ABTS (μmol TE/g DP)	FRAP (μmol Fe^2+^/g DP)
Plant										
Tarragon	23.1 ± 1.34 ^bc^	96.9 ± 28.7 ^a^	132 ± 33.0 ^bc^	4.19 ± 0.83 ^c^	8.05 ± 0.78 ^c^	20.0 ± 0.95 ^e^	17.6 ± 2.44 ^b^	61.8 ± 6.59 ^c^	107 ± 6.07 ^c^	191 ± 11.5 ^e^
Spearmint	21.5 ± 1.33 ^d^	92.6 ± 23.8 ^a^	149 ± 35.0 ^ab^	8.91 ± 0.81 ^b^	30.5 ± 1.23 ^b^	44.0 ± 3.16 ^c^	13.7 ± 1.39 ^cd^	259 ± 14.4 ^b^	361 ± 20.4 ^b^	722 ± 31.0 ^b^
Lemon balm	26.2 ± 1.24 ^a^	96.1 ± 14.8 ^a^	209 ± 33.1 ^a^	10.4 ± 0.77 ^a^	45.6 ± 4.86 ^a^	74.4 ± 3.90 ^a^	22.0 ± 1.76 ^a^	345 ± 11.0 ^a^	507 ± 28.6 ^a^	1013 ± 75.5 ^a^
Basil	22.2 ± 1.87 ^cd^	68.9 ± 16.8 ^b^	108 ± 22.1 ^d^	6.02 ± 0.94 ^c^	16.2 ± 1.07 ^c^	26.9 ± 2.07 ^d^	11.8 ± 1.21 ^d^	149 ± 12.0 ^c^	194 ± 12.6 ^c^	376 ± 28.0 ^d^
French lavender	25.2 ± 0.78 ^ab^	43.5 ± 6.60 ^c^	115 ± 9.18 ^cd^	10.7 ± 1.54 ^a^	32.2 ± 0.94 ^b^	43.1 ± 2.45 ^c^	21.6 ± 0.88 ^a^	241 ± 20.5 ^b^	326 ± 25.4 ^b^	614 ± 51.4 ^c^
Sage	22.4 ± 0.41 ^cd^	59.8 ± 12.4 ^bc^	99.9 ± 13.4 ^d^	8.98 ± 1.16 ^b^	30.9 ± 2.18 ^b^	49.5 ± 3.87 ^b^	16.5 ± 1.65 ^bc^	265 ± 13.8 ^b^	358 ± 23.1 ^b^	752 ± 44.5 ^b^
Method										
Solid-liquid	24.8 ± 0.55 ^a^	80.8 ± 9.85 ^a^	163 ± 16.6 ^a^	6.15 ± 0.46 ^b^	26.3 ± 2.39 ^b^	44.5 ± 3.46 ^a^	18.0 ± 0.92 ^a^	216 ± 17.0 ^a^	310 ± 24.6 ^a^	579 ± 47.8 ^b^
Soxhlet	21.4 ± 0.91 ^b^	69.7 ± 12.7 ^b^	93.6 ± 8.46 ^b^	11.3 ± 0.71 ^a^	28.6 ± 2.83 ^a^	40.7 ± 3.68 ^b^	16.1 ± 1.44 ^b^	226 ± 20.5 ^a^	307 ± 28.7 ^a^	660 ± 65.7 ^a^
Solvent										
Water	21.1 ± 0.73 ^a^	28.1 ± 4.41 ^b^	86.6 ± 12.2 ^b^	8.43 ± 0.70 ^a^	25.5 ± 2.23 ^b^	44.3 ± 3.54 ^a^	18.1 ± 1.29 ^a^	212 ± 16.1 ^a^	291 ± 23.2 ^b^	577 ± 49.4 ^b^
EtOH 70%	25.8 ± 0.50 ^b^	124 ± 8.06 ^a^	184 ± 14.5 ^a^	7.97 ± 0.75 ^b^	29.0 ± 2.88 ^a^	41.7 ± 3.67 ^b^	16.3 ± 0.95 ^b^	229 ± 20.6 ^b^	327 ± 29.0 ^a^	646 ± 60.2 ^a^
Main effects										
Plant	***	***	***	***	***	***	***	***	***	***
Method	***	***	***	***	***	**	*	.	NS	***
Solvent	***	***	***	**	***	*	*	**	***	***
Interactions										
Plant × Method	**	***	***	***	***	***	***	.	*	*
Plant × Solvent	***	***	***	***	***	***	**	***	***	***
Method × Solvent	***	NS	***	***	***	***	NS	***	***	***
Plant × Method × Solvent	*	**	***	***	***	**	.	*	*	**

DP: dry plant; Mean values with different superscript letters in a column are significantly different. “NS”: *p* < 1; “.”: *p* < 0.1; “*”: *p* < 0.05; “**”: *p* < 0.01; “***”: *p* < 0.001.

**Table 2:** Identification and quantification of phenolic compounds present in the extracts produced.

Phenolic Compound (mg/L Extract)			Chlorogenic Acid	Vanillic Acid	Syringic Acid	Cinnamic Acid	*p*-Coumaric Acid + Epicatechin	*o*-Coumaric Acid	Rosmarinic Acid	Ellagic Acid	Naringin	Hesperidin	Kaempferol	Resveratrol	Ferulic Acid	Quercetin	3,4HBA
Soxhlet	H_2_O	Tarragon	27.4 ± 0.79	nd	nd	9.61 ± 1.11	165 ± 10.0	62.6 ± 6.38	45.9 ± 2.60	645 ± 31.0	270 ± 18.9	99.4 ± 7.57	nd	15.5 ± 2.74	111 ± 2.83	3.31 ± 0.64	9.16 ± 0.56
		Spearmint	8.77 ± 0.22	nd	nd	nd	nd	nd	324 ± 32.4	279 ± 21.9	55.7 ± 9.32	561 ± 45.9	nd	25.7 ± 2.12	55.1 ± 1.24	5.21 ± 0.26	nd
		Lemon balm	12.2 ± 1.76	nd	nd	nd	nd	nd	448 ± 109	373 ± 179	105 ± 31.1	901 ± 232	nd	59.3 ± 21.8	18.2 ± 6.79	12.6 ± 5.26	nd
		Basil	nd	nd	nd	nd	12.3 ± 1.72	nd	128 ± 2.73	420 ± 126	69.6 ± 3.30	206 ± 1.53	nd	5.94 ± 5.52	51.6 ± 2.89	4.13 ± 0.07	nd
		French lavender	nd	nd	nd	nd	33.4 ± 12.1	nd	198 ± 0.75	75.9 ± 5.61	71.8 ± 10.8	85.0 ± 13.5	93.7 ± 6.69	103 ± 6.35	74.3 ± 12.1	10.3 ± 2.01	nd
		Sage	nd	nd	nd	nd	184 ± 16.6	nd	435 ± 41.8	587 ± 423	523 ± 33.8	900 ± 71.4	nd	4.92 ± 0.62	161 ± 11.7	5.53 ± 0.04	nd
	EtOH 70%	Tarragon	nd	nd	nd	nd	93.3 ± 0.75	2.45 ± 0.25	38.9 ± 1.44	472 ± 41.6	133 ± 8.66	61.4 ± 1.37	nd	16.3 ± 0.42	68.6 ± 2.55	5.79 ± 2.04	1.27 ± 0.14
		Spearmint	nd	nd	nd	nd	nd	nd	555 ± 30.7	416 ± 32.4	92.7 ± 9.53	1131 ± 63.2	63.2 ± 2.70	68.1 ± 5.23	49.1 ± 4.78	19.4 ± 1.87	nd
		Lemon balm	nd	nd	nd	nd	nd	nd	679 ± 61.8	238 ± 0.48	93.5 ± 2.66	1369 ± 105	63 ± 3.37	97.9 ± 7.57	0.81 ± 0.06	8.60 ± 0.65	nd
		Basil	nd	nd	nd	nd	nd	nd	143 ± 5.48	150 ± 5.07	34.3 ± 0.72	242 ± 10.4	nd	10.8 ± 1.21	9.00 ± 0.05	6.39 ± 2.55	nd
		French lavender	nd	nd	nd	nd	nd	nd	244 ± 27.9	554 ± 69.2	116 ± 0.61	495 ± 45.5	77.1 ± 8.35	127 ± 7.71	88.8 ± 10.1	27.2 ± 6.52	nd
		Sage	nd	nd	nd	nd	4.14 ± 0.84	nd	523 ± 3.85	249 ± 11.0	537 ± 8.05	996 ± 113	98.8 ± 4.43	44.5 ± 5.01	163 ± 3.85	33.0 ± 4.47	nd
Solid-liquid	H_2_O	Tarragon	nd	nd	nd	25.0 ± 3.32	57.6 ± 4.27	44.2 ± 5.08	341 ± 28.8	42.9 ± 5.33	267 ± 32.5	43.3 ± 6.59	nd	33.6 ± 1.21	34.9 ± 2.51	15.9 ± 1.01	nd
		Spearmint	nd	nd	nd	150 ± 3.79	53.7 ± 6.40	20.5 ± 0.14	204 ± 53.2	28.3 ± 2.58	22.8 ± 0.53	110 ± 26.6	nd	75.2 ± 8.25	42.8 ± 1.28	71.4 ± 10.9	nd
		Lemon balm	nd	nd	nd	56.3 ± 8.94	nd	99.9 ± 59.9	129 ± 11.9	82.1 ± 3.43	116 ± 5.43	31.6 ± 0.14	nd	90.9 ± 8.22	80.7 ± 1.71	43.1 ± 0.81	nd
		Basil	nd	17.2 ± 0.30	10.5 ± 0.24	80.5 ± 1.32	103 ± 11.8	33.9 ± 1.35	274 ± 10.9	40.2 ± 3.54	287 ± 1.17	144 ± 9.77	nd	46.4 ± 0.95	39.0 ± 0.17	34.2 ± 0.62	nd
		French lavender	nd	nd	nd	80.5 ± 6.93	nd	30.9 ± 6.91	120 ± 42.9	39.2 ± 0.32	63.5 ± 27.0	116 ± 5.51	nd	95.2 ± 2.01	64.5 ± 11.1	29.5 ± 0.39	nd
		Sage	nd	12.4 ± 0.05	9.33 ± 0.63	53.3 ± 1.26	nd	36.7 ± 0.79	173 ± 101	43.3 ± 14.5	78.0 ± 12.0	279 ± 30.8	nd	93.9 ± 3.53	35.9 ± 3.55	54.6 ± 7.35	nd
	EtOH 70%	Tarragon	nd	9.92 ± 0.03	nd	37.2 ± 4.66	nd	33.2 ± 4.99	355 ± 5.15	38.5 ± 7.58	123 ± 0.52	292 ± 5.89	2.73 ± 0.38	95.6 ± 2.38	47.0 ± 8.11	33.7 ± 0.29	nd
		Spearmint	nd	nd	nd	280 ± 10.6	69.0 ± 3.17	27.8 ± 0.61	333 ± 57.3	28.6 ± 0.40	62.9 ± 2.16	223 ± 12.7	1.13 ± 0.32	111 ± 7.50	60.7 ± 13.9	55.7 ± 2.67	nd
		Lemon balm	71.2 ± 0.48	nd	nd	487 ± 15.8	123 ± 1.36	111 ± 92.4	185 ± 27.2	50.5 ± 4.25	894 ± 51.8	3.71 ± 3.34	nd	126 ± 9.33	108 ± 35.9	41.2 ± 0.11	nd
		Basil	64.9 ± 1.25	12.9 ± 0.18	nd	79.9 ± 4.15	84.4 ± 10.7	25.5 ± 2.01	292 ± 4.31	31.0 ± 2.62	65.1 ± 7.86	188 ± 13.5	nd	85.4 ± 1.27	50.2 ± 7.47	18.1 ± 0.21	nd
		French lavender	32.2 ± 0.22	nd	nd	121 ± 2.21	68.5 ± 0.85	31.6 ± 0.59	127 ± 18.4	49.9 ± 14.7	77.8 ± 2.31	123 ± 8.73	nd	96.2 ± 0.86	55.1 ± 0.56	27.3 ± 0.03	nd
		Sage	nd	nd	nd	485 ± 66.3	119 ± 3.22	94.9 ± 4.13	170 ± 13.6	52.2 ± 9.61	279 ± 16.1	805 ± 40.0	nd	200 ± 13.0	78.5 ± 4.35	129 ± 4.76	16.0 ± 0.30

3,4HBA: 3,4-dihydroxybenzoic acid; nd: not detected.

## References

[1] Silva B.N., Cadavez V., Ferreira-Santos P., Alves M.J., Ferreira I.C.F.R., Barros L., Teixeira J.A., Gonzales-Barron U. (2021). Chemical Profile and Bioactivities of Extracts from Edible Plants Readily Available in Portugal. Foods.

