# Supplementary material for: Chemical Profile and Bioactivities of Extracts from Edible Plants Readily Available in Portugal
Source: Foods. 2021 Mar 22;10(3):673. doi: 10.3390/foods10030673 (PMC8004287; doi:10.3390/foods10030673)
Supplement: Supplementary file 1 [file foods-10-00673-s001.zip › foods-1153849-supplementary.pdf]

Table S1. Limit of detection (LOD), limit of quantification (LOQ) and coefficient of linear correlation (R^2) of the different phenolic compounds tested.

Phenolic compound	LOD (mg/L)	LOQ (mg/L)	R^2
Chlorogenic acid	17.71	53.67	0.9988
Vanillic acid	9.57	29.00	0.9996
Syringic acid	2.24	16.80	0.9999
Cinnamic acid	13.99	42.40	0.9998
p-coumaric acid + epicatechin	12.78	38.71	0.9986
o-coumaric acid	11.75	35.60	0.9988
Rosmarinic acid	14.42	43.71	0.9970
Ellagic acid	30.65	92.88	0.9912
Naringin	9.89	29.96	0.9992
Hesperidin	36.87	111.7	0.9996
Kaempferol	55.48	107.5	0.9960
Resveratrol	32.95	99.85	0.9909
Ferulic acid	31.19	94.53	0.9916
Quercetin	21.34	64.67	0.9961
3,4-Dihydroxybenzoic acid	9.60	29.07	0.9993

Table S2. Extraction yield, chemical profile and antioxidant activity of the extracts produced (mean ± standard deviation).

			Yield (%)	Ch-a (µg/g DP)	Ch-b (µg/g DP)	TProtein (µg BSAE/g DP)	TFC (mg CE/g DP)	TPC (mg GAE/g DP)	Carbohydr. (µg GE/g DP)	DPPH (µmol TE/g DP)	ABTS (µmol TE/g DP)	FRAP (µmol Fe^{2+}/g DP)
Soxhlet	H_2O	Tarragon	26.8 ± 0.55	11.9 ± 0.1	40.9 ± 0.33	5.54 ± 0.07	8.78 ± 0.18	24.3 ± 0.41	5.94 ± 0.04	94.9 ± 1.50	130 ± 4.48	232 ± 3.71
		Spearmint	23.6 ± 0.90	24.0 ± 0.17	72.9 ± 0.24	13.1 ± 0.20	32.8 ± 1.62	44.8 ± 2.29	8.91 ± 0.64	268 ± 0.50	379 ± 6.22	777 ± 8.57
		Lemon balm	25.4 ± 1.40	25.3 ± 2.50	81.8 ± 7.15	11.3 ± 0.76	53.5 ± 1.51	77.8 ± 9.56	29.0 ± 2.79	363 ± 19.51	533 ± 31.1	1182 ± 126
		Basil	26.1 ± 1.70	13.4 ± 4.50	44.2 ± 12.7	10.7 ± 0.34	18.7 ± 1.03	33.4 ± 3.38	14.8 ± 4.18	193 ± 1.20	227 ± 3.48	509 ± 19.7
		French lavender	25.5 ± 2.10	34.0 ± 10.5	106 ± 23.8	13.5 ± 0.34	34.8 ± 4.44	56.5 ± 4.06	20.5 ± 2.41	293 ± 37.5	389 ± 51.9	818 ± 65.5
		Sage	22.5 ± 0.10	19.5 ± 0.30	62.2 ± 1.10	11.7 ± 0.07	34.8 ± 0.57	50.8 ± 0.83	23.0 ± 2.55	266 ± 0.50	383 ± 4.72	791 ± 35.1
	EtOH 70%	Tarragon	16.3 ± 0.40	194 ± 0.44	175 ± 0.12	7.22 ± 0.48	12.3 ± 0.74	15.9 ± 0.71	13.1 ± 0.37	62.9 ± 1.08	102 ± 19.0	204 ± 19.1
		Spearmint	15.0 ± 0.25	177 ± 5.25	137 ± 2.32	9.85 ± 0.08	26.7 ± 0.62	30.7 ± 0.60	8.70 ± 0.96	232 ± 12.8	289 ± 10.1	689 ± 36.3
		Lemon balm	20.6 ± 0.05	86.1 ± 5.56	99.7 ± 12.7	12.5 ± 1.41	47.9 ± 1.07	54.5 ± 5.56	15.6 ± 0.27	360 ± 2.36	492 ± 8.13	1094 ± 22.9
		Basil	13.4 ± 0.15	112 ± 4.58	84.9 ± 1.69	7.57 ± 0.46	10.8 ± 0.02	16.6 ± 0.19	10.7 ± 0.74	85.4 ± 0.97	123 ± 0.99	256 ± 1.52
		French lavender	21.4 ± 0.40	55.0 ± 9.31	136 ± 31.8	18.2 ± 1.86	30.1 ± 0.43	40.4 ± 2.37	21.2 ± 1.05	213 ± 41.6	277 ± 52.9	511 ± 80.6
		Sage	20.6 ± 0.50	83.6 ± 10.3	83.1 ± 18.6	14.2 ± 1.45	32.5 ± 1.71	43.3 ± 2.50	21.2 ± 1.03	281 ± 23.8	357 ± 27.6	857 ± 66.7
Solid-liquid	H_2O	Tarragon	26.2 ± 1.87	11.0 ± 0.99	34.7 ± 3.20	0.63 ± 0.15	6.59 ± 0.62	19.3 ± 0.85	24.4 ± 3.27	57.8 ± 1.54	87.0 ± 2.24	143 ± 2.72
		Spearmint	25.4 ± 2.12	20.1 ± 5.84	55.2 ± 13.4	7.84 ± 0.44	28.5 ± 3.81	40.4 ± 2.47	17.4 ± 0.37	220 ± 28.0	324 ± 30.9	621 ± 65.6
		Lemon balm	31.0 ± 0.22	95.4 ± 11.2	271 ± 26.3	11.5 ± 1.56	25.5 ± 7.15	81.9 ± 2.90	24.8 ± 2.29	303 ± 29.0	410 ± 75.3	693 ± 97.7
		Basil	27.9 ± 0.32	22.6 ± 0.99	67.5 ± 2.26	4.97 ± 0.18	18.9 ± 0.56	30.9 ± 1.48	12.1 ± 2.76	157 ± 10.6	197 ± 129	400 ± 9.76
		French lavender	25.9 ± 0.35	20.5 ± 0.29	87.7 ± 1.89	8.32 ± 0.21	31.7 ± 0.65	40.9 ± 1.30	21.4 ± 3.68	183 ± 54.8	262 ± 63.3	478 ± 112
		Sage	22.1 ± 0.22	26.6 ± 1.33	77.6 ± 3.13	7.15 ± 0.51	21.6 ± 0.88	37.6 ± 4.54	13.3 ± 2.07	210 ± 9.79	265 ± 3.16	562 ± 14.7
	EtOH 70%	Tarragon	22.2 ± 0.42	175 ± 9.58	260 ± 1.21	4.82 ± 0.21	6.22 ± 0.26	20.6 ± 1.28	21.4 ± 1.26	43.0 ± 14.1	114 ± 3.71	203 ± 6.45
		Spearmint	20.6 ± 0.90	155 ± 0.06	303 ± 10.6	6.55 ± 0.55	33.4 ± 1.95	56.1 ± 3.52	16.7 ± 1.61	312 ± 21.3	434 ± 31.0	808 ± 57.2
		Lemon balm	25.8 ± 0.52	151 ± 0.31	306 ± 2.84	7.40 ± 0.38	59.1 ± 4.62	77.7 ± 5.38	18.9 ± 2.35	365 ± 0.98	596 ± 15.4	1166 ± 34.3
		Basil	19.6 ± 0.91	124 ± 0.58	207 ± 4.68	2.95 ± 0.16	15.3 ± 1.26	25.6 ± 1.29	10.3 ± 3.95	153 ± 10.0	217 ± 6.84	342 ± 11.7
		French lavender	27.3 ± 0.71	65.4 ± 4.28	133 ± 8.87	6.16 ± 0.16	32.2 ± 2.28	38.1 ± 2.93	22.7 ± 2.66	283 ± 11.0	381 ± 15.4	682 ± 39.8
		Sage	23.9 ± 0.40	104 ± 3.17	158 ± 8.87	5.49 ± 0.17	36.7 ± 2.89	64.7 ± 4.91	12.3 ± 0.82	310 ± 7.84	437 ± 11.4	846 ± 44.7
